# Trends over Time in Adolescent Emotional Wellbeing in the Netherlands, 2005-2017: Links with Perceived Schoolwork Pressure, Parent-Adolescent Communication and Bullying Victimization

**DOI:** 10.1007/s10964-020-01280-4

**Published:** 2020-07-23

**Authors:** M. E. De Looze, A. P. Cosma, W. A. M. Vollebergh, E. L. Duinhof, S. A. de Roos, S. van Dorsselaer, M. J. H. van Bon-Martens, R. Vonk, G. W. J. M. Stevens

**Affiliations:** 1 Department of Interdisciplinary Social Science, Faculty of Social and Behavioural Sciences, Utrecht University, P.O. Box 80.140, 3508 TC Utrecht, The Netherlands; 2 The Netherlands Institute for Social Research (SCP), P.O. Box 16164, 2500 BD Den Haag, The Netherlands; 3 Netherlands Institute of Mental Health and Addiction, P.O. Box 725, 3500 AS Utrecht, The Netherlands; 4 National Institute for Public Health and the Environment (RIVM), Center for Health and Society, P.O. Box 1, 3720 BA Bilthoven, The Netherlands

**Keywords:** Emotional wellbeing, Psychosomatic complaints, Adolescence, Schoolwork pressure, Time trends, Netherlands

## Abstract

In some Scandinavian countries, the United Kingdom and the United States, there is evidence of a dramatic decline in adolescent emotional wellbeing, particularly among girls. It is not clear to what extent this decline can be generalised to other high-income countries. This study examines trends over time (2005-2009-2013-2017) in adolescent wellbeing in the Netherlands, a country where young people have consistently reported one of the highest levels of wellbeing across Europe. It also assesses parallel changes over time in perceived schoolwork pressure, parent-adolescent communication, and bullying victimization. Data were derived from four waves of the nationally representative, cross-sectional Dutch Health Behaviour in School-aged Children study (*N* = 21,901; 49% girls; *M*_age_ = 13.78, SD = 1.25). Trends in emotional wellbeing (i.e., emotional symptoms, psychosomatic complaints, life satisfaction) were assessed by means of multiple regression analyses with survey year as a predictor, controlling for background variables. Emotional wellbeing slightly declined among adolescent boys and girls between 2009 and 2013. A substantial increase in perceived schoolwork pressure was associated with this decline in emotional wellbeing. Improved parent-adolescent communication and a decline in bullying victimization may explain why emotional wellbeing remained stable between 2013 and 2017, in spite of a further increase in schoolwork pressure. Associations between emotional wellbeing on the one hand and perceived schoolwork pressure, parent-adolescent communication, and bullying victimization on the other were stronger for girls than for boys. Overall, although increasing schoolwork pressure may be one of the drivers of declining emotional wellbeing in adolescents, in the Netherlands this negative trend was buffered by increasing support by parents and peers. Cross-national research into this topic is warranted to examine the extent to which these findings can be generalised to other high-income countries.

## Introduction

Adolescent emotional wellbeing is a public health concern worldwide. Since the early 21st century, declines in adolescent emotional wellbeing (i.e., lower life satisfaction, more emotional symptoms, and more psychosomatic health complaints) have been observed in high-income countries such as Denmark, Sweden, Iceland, the UK, and the United States (Bor et al. 2014; Potrebny et al. 2017; Twenge et al. 2018). Important explanations of this decline in wellbeing include an increase in perfectionism among young people (Curran and Hill 2019) and increasing worries about schoolwork and about the future, such as the fear that they will not find a job or earn enough money for a living (e.g., The Children’s Society 2017). While the decline in emotional wellbeing among youth has aroused much attention by the general public, practitioners, policymakers and politicians, it is not clear to what extent the decline in adolescent emotional wellbeing can be generalised across countries. The aim of this study was to examine recent trends (2005–2017) in emotional wellbeing among boys and girls in the Netherlands, and to analyse to what extent these trends may be associated with recent developments in three important social domains of adolescent life: the school, peer and family context.

The Netherlands are an interesting case study when it comes to the wellbeing of young people. Since the early 21st century, young people in the Netherlands have consistently reported among the highest levels of wellbeing across Europe (Currie et al. 2008a; 2012; Inchley et al. 2016). Trends over time have been remarkably stable; yet, between 2009 and 2013, there was a small increase in emotional problems (Duinhof et al. 2015) and a slight decrease in life satisfaction (De Looze et al. 2014), especially among girls. Research explaining this small decline in wellbeing is scarce.

From an ecological point of view (Bronfenbrenner and Morris 2006), young people’s development is embedded in different layers of the environmental context. During adolescence, three of the most important social contexts are the school, peer, and family context. Protective factors in these contexts, such as positive family relations, predict high emotional wellbeing, while stressors, such as negative school experiences, predict low emotional wellbeing (Chu et al. 2010; Viner et al. 2012). Youth in the Netherlands have typically reported very favourable indications in all three domains of life, but some of these appear to be changing in a less favourable direction.

As to the school-context, one of the strongest predictors of emotional wellbeing is perceived schoolwork pressure (Wiklund et al. 2012). Young people who feel pressured by increasing demands, competition or having to succeed, typically report lower emotional wellbeing, compared to those who do not (Wiklund et al. 2012). For years, adolescents in the Netherlands have reported among the lowest levels of perceived schoolwork pressure, as compared to other high-income countries (Currie et al. 2008a; 2012; Inchley et al. 2016). However, in recent years, perceptions of schoolwork pressure appear to be changing. While in 2005 only 19% of Dutch adolescents reported high schoolwork pressure, this percentage increased to 28% in 2013 (Stevens et al. 2018). Moreover, in a qualitative study, young people (age 15–23 years old) in the Netherlands indicated experiencing a high pressure to be perfect (both regarding their school work and personal life); a fear to disappoint others, particularly their parents; and to believe that only hard work, perseverance and success are rewarded in the current society and are, thus, needed to succeed in life (Schoemaker et al. 2019). The increase in perceived schoolwork pressure among adolescents in the Netherlands may be the result of increasingly strict requirements needed to graduate in the Dutch school system (since 2011; Examenblad 2018). However, the increasing trend in perceived schoolwork pressure is also in line with an international trend towards increased perfectionism and ambitiousness among adolescents in high-income countries (Curran and Hill 2019). Potentially, the increase in perceived schoolwork pressure and ambitiousness may be linked to the historical fact that youth nowadays may be the first generation to experience downward mobility (i.e., the situation in which people find themselves in a lower social class than the one in which they were born; Janssen et al. 2018). Young people these days enter a crowded professional job market and are often forced to accept lower-level positions, also in the Netherlands (Janssen et al. 2018). All of these developments may have increased young people’s feeling that they *have* to do well at school, which may have given rise to a decline in emotional wellbeing.

In contrast, in the family- and peer domain the trends seem to be more favourable, with Dutch adolescents systematically scoring relatively positively, as compared to other countries. For instance, the percentage of Dutch youth who indicated to be bullied at least two times per month, was around 7% in 2005, 2009 and 2013 (Stevens et al. 2018). As such, Dutch youth scored in the lower 1/3 percentile of Europe (Currie et al. 2008a; 2012; Inchley et al. 2016). Moreover, the Netherlands have consistently top ranked when it comes to the family communication, with 77% of adolescents being able to easily talk with their fathers and 88% with their mothers about things that bother them in 2013 (De Looze et al. 2014). Family interactions present opportunities for parents to shape coping and positive health behaviours and enable adolescents to express their concerns and feel valued, and therefore easy parent-adolescent communication is an important predictor of adolescent mental health (e.g., Elgar et al. 2013; Repetti et al. 2002). Taken together, while the trend towards increasing perceived schoolwork pressure suggests a further decline in adolescent emotional wellbeing after 2013, the stable positive social relationships with parents and peers that adolescents in the Netherlands typically report may serve as a buffer against such a decline in emotional wellbeing.

When assessing (trends in) young people’s emotional wellbeing, it is imperative to analyse gender differences. Across a wide variety of wellbeing indicators, girls consistently report lower emotional wellbeing than boys (De Looze et al. 2018; Duinhof et al. 2015; Inchley et al. 2016) and there is some international research to suggest that declines in wellbeing in the last decade are stronger among girls than boys (Bor et al. 2014; Twenge 2017). Girls have also been found to report higher levels of schoolwork pressure, less easy communication with parents, and higher peer support than boys (Stevens et al. 2018). Moreover, some models (e.g., Hyde et al. 2008) suggest that biological, affective and cognitive vulnerabilities in females interact with negative life-events, in such a way that social stressors (e.g., perceived schoolwork pressure) have a larger impact on the emotional wellbeing of girls than boys (Hankin et al. 2007). As such, girls appear to be most at risk for a potential decline in emotional wellbeing. Gender differences, consequently, form a red thread throughout this study.

## Current Study

With declines being observed in emotional wellbeing among youth—in particular in girls—in some high-income countries, societal concerns about young people’s emotional wellbeing are on the rise. The current study examines whether the decline in emotional wellbeing also applies to youth in the Netherlands and aims to place potential trends in the context of school, family and peer relations. It was predicted that adolescent emotional wellbeing declined (Hypothesis 1); that this decline occurred in parallel with an increase in perceived schoolwork pressure (Hypothesis 2); and that ongoing high levels of parent-adolescent communication and low levels of bullying victimization functioned as protective factors against this decline in emotional well-being (Hypothesis 3). Finally, emotional well-being was expected to have declined stronger among girls, as compared to boys. Moreover, associations between emotional wellbeing on the one hand and perceived schoolwork pressure, parent-adolescent communication and bullying victimization on the other were expected to be stronger for girls, as compared to boys (Hypothesis 4).

## Methods

### Study Sample and Procedures

Four data waves (2005, 2009, 2013, 2017) of the nationally representative HBSC study in the Netherlands were used (*N* = 21,901; De Looze et al. 2014; Stevens et al. 2018; Van Dorsselaer et al. 2007; 2010). In the Netherlands, the HBSC study is carried out by Utrecht University, the Netherlands Institute for Mental Health and Addiction (Trimbos institute) and the Netherlands Institute for Social Research (SCP). The sampling and survey procedures for the different survey waves were identical and the present examination had a repeated cross-sectional design. The study included data from adolescents aged 11 to 16 attending the first four classes of general secondary education. The samples were obtained using a two-stage random sampling procedure. First, schools were stratified and drawn proportionally according to the level of urbanisation, based on a national file of regular secondary schools, provided by the ministry of Education, Culture and Science. Pupils with major learning disorders or psychiatric problems are in special education and are excluded from the sample. Second, within each school two to five classes (depending on school size) were selected randomly from a list of all classes provided by each participating school. Within the selected classes, all students were drawn as a single cluster. The school level response rates ranged from 37% (2013/2017) to 48% (2009). The reasons for non-response at the school level were mainly related to (frequently being approached for) participation in other research. Research assistants administered self-report paper-and pencil questionnaires in classroom setting (2005, 2009, and 2013), whereas in the last survey round (2017) data were collected via web based questionnaires. Respondents were assured of the anonymity and confidentiality of their responses. Participant non-response rates were low (<10%) and mainly because of illness.

Sample sizes ranged from *N* = 5187 (2005) to *N* = 5834 (2017). Across all survey years, 51% of the participants were boys. The average age of the participants ranged from 13.72 (*SD* = 1.25) (2017) to *M*_age_ = 13.81 (SD = 1.26) (2005).

### Measures

#### Emotional wellbeing

##### Emotional symptoms

The Emotional Symptoms subscale (including 5 items) of the Strengths and Difficulties Questionnaire (SDQ) was used to assess emotional symptoms. The SDQ is a screening questionnaire that asks adolescents to report on their behaviours and emotions in the past 6 months (Goodman et al. 1998). Items (e.g., “I worry a lot”; “I am often unhappy, down-hearted or tearful”) were scored on a three-point Likert scale “not true”, “somewhat true”, “certainly true”. The response were summed up with higher scores indicating more emotional symptoms. The emotional symptoms subscale of the SDQ is measurement invariant over time and between boys and girls, adolescents with a native Dutch versus immigrant background, and vocational and academic educated adolescents (Duinhof et al. 2015). In our sample, this subscale had an acceptable reliability (Cronbach’s alpha = 0.69).

##### Psychosomatic complaints

The HBSC Symptom Checklist, a non-clinical measure consisting of eight health complaint items was used to measure psychological and somatic health complaints (e.g., head ache; feeling low). Participants had to indicate how often they experienced these symptoms over the last six months. Response categories were: (1) “about every day”, (2) “more than once a week”, (3) “about every week”, (4) “about every month” and (5) “rarely or never”. To obtain a meaningful interpretation, prior to creating a mean score the items were reverse coded (scale 0 to 4). This instrument has adequate test-retest reliability and convergent validity (Haugland and Wold 2001). In our sample this instrument showed good reliability (Cronbach’s alpha = 0.80).

##### Life satisfaction

Participants rated their life satisfaction on the Cantril ladder, a scale ranging from the worst possible life (0) to the best possible life (10) (Cantril 1965). The Cantril ladder is a reliable and valid instrument for wellbeing among adolescents (Jovanovic 2016; Levin and Currie 2014).

#### Explanatory variables

##### Gender

Adolescents were asked to indicate whether they were a boy or a girl. Boys were set as the reference group.

##### Perceived schoolwork pressure

Participants responded to the question “How pressured do you feel by the schoolwork you have to do?”. The response options available were (1) “not at all”, (2) “a little”, (3) “some”, and (4) “a lot”. This is often considered a measure of school-related stress, and associations have been documented with risk behaviours, frequent health complaints, psychological complaints, and poor mental health (e.g., Ottova et al. 2012).

##### Perceived communication with mother and father

Participants were asked how easy it was to talk to their mother or father about issues that were bothering them. Response options ranged from (1) “very easy,” to (4) “very difficult,” and (5) “don’t have or don’t see this person.”. For the purpose of this study, the scale has been recoded to 1 (very difficult) to 4 (very easy). Those respondents that indicated that they do not have or see that person were removed from the analysis. These percentages ranged from 4.9% (2009) to 6.3% (2013) for communication with father, and from 1.4% (2017) to 2.9% (2005) for communication with mother. This measure has been used for cross-national comparisons within HBSC study since 1994 (Inchley et al. 2018).

##### Bullying victimization

An adapted version of the Olweus bullying victimization questionnaire (Olweus 1996) was used. After reading a definition of bullying, the participants were asked to indicate if they have been bullied at school in the past couple of months with the following response options: (1) “I haven’t been bullied”, (2) “only 1–2 times”, (3) “2–3 times a month”, (4) “About once a week”, (5) “Several times a week”. Following HBSC international recommendation (Inchley et al. 2018), the measure was amended in 2017 when the reference of “only” was excluded from the second response option and the initial definition was slightly re-worded in order to be shorter and more child friendly.

#### Control variables

##### Age

Adolescents were asked to indicate their month and year of birth. Using the date of the data collection, their age was calculated.

##### Immigration background

Ethnicity was based on the country of birth of adolescents and their parents. If at least one parent was born abroad, adolescents were identified as having a non-native background.

##### Family structure

Family structure was determined by a series of binary variables derived from three related questions. The first question asks who resides in the home where the respondent lives all or most of the time, including father, mother, stepfather and stepmother. The second question asks if the respondent has another home or another family and how often he or she stays there (half the time, regularly but less than half the time, sometimes, hardly ever). The third question asks who lives in the second home. Based on these items, a dichotomous variable was created, distinguishing between adolescents who lived with both biological parents in the primary household (1) and those who did not (0).

##### Educational track

The Dutch educational system has four educational tracks, ranging from vocational training (VMBO-b) to higher academic education (VWO). Adolescents were asked to indicate their educational track in the questionnaire.

##### Family affluence

Family affluence was assessed through the revised Family Affluence Scale (FAS II) (Currie et al. 2008b). This scale includes 4 items related to the material conditions in the participant household (i.e., ownership of a car, own bedroom, number of computers, and abroad holiday frequency during the last year). Individual responses were scored and summed to provide summary scores ranging from 0 to 9 with higher values indicating higher levels of family affluence.

### Analytic Strategy

All analyses were performed using statistic software package SPSS v24. To assure national representativeness, data were weighted for educational level, grade, gender, and urbanicity. All analyses were controlled for age, immigration background, family structure, educational track and family affluence. To correct for the large datasets and the large amount of tests, associations and interaction effects were considered significant only if *p* < 0.001. The missing rates were low (below 4%) for all variables apart from communication with father (6%). Given the low levels of missing data, all regression models used the list-wise deletion approach.

First, to describe trends in emotional wellbeing, the estimated means were calculated per survey year. Subsequently, trend analyses were conducted using multiple regression analysis. To test the extent to which perceived schoolwork pressure, parent-adolescent communication and bullying victimization accounted for the change over time in adolescent emotional wellbeing, emotional wellbeing was modelled as a function of survey year, adjusting for demographic factors (Model 1). Next, the explanatory variables were added to model 1, first individually, then collectively. Attenuation of the regression coefficient for year, which was tested using Sobel tests, would indicate that the explanatory variable (partially) accounted for the trend over time in emotional wellbeing. Sobel tests are typically conducted for testing mediation effects. By conducting Sobel tests in the relationship between survey year (independent variable) and wellbeing (dependent variable) via, for example, perceived schoolwork pressure, it is tested whether a change over time in schoolwork pressure explains a change over time in wellbeing. While no causative conclusions can be drawn in the current study due to the cross-sectional nature of the data, the Sobel test indicates whether changes over time in perceived schoolwork pressure are significantly associated with changes over time in wellbeing, and whether perceived schoolwork pressure and wellbeing are significantly related to one another.

Finally, to test whether trends in emotional wellbeing differed between boys and girls, interaction analyses (survey year x gender) were added to Model 1. Additionally, interaction analyses (explanatory variable × gender) were added to test whether the association between emotional wellbeing on the one hand and perceived schoolwork pressure, parent-adolescent communication, and bullying victimization on the other, differed between boys and girls.

## Results

### Emotional Wellbeing: Descriptive Trends

**Fig. 1:**
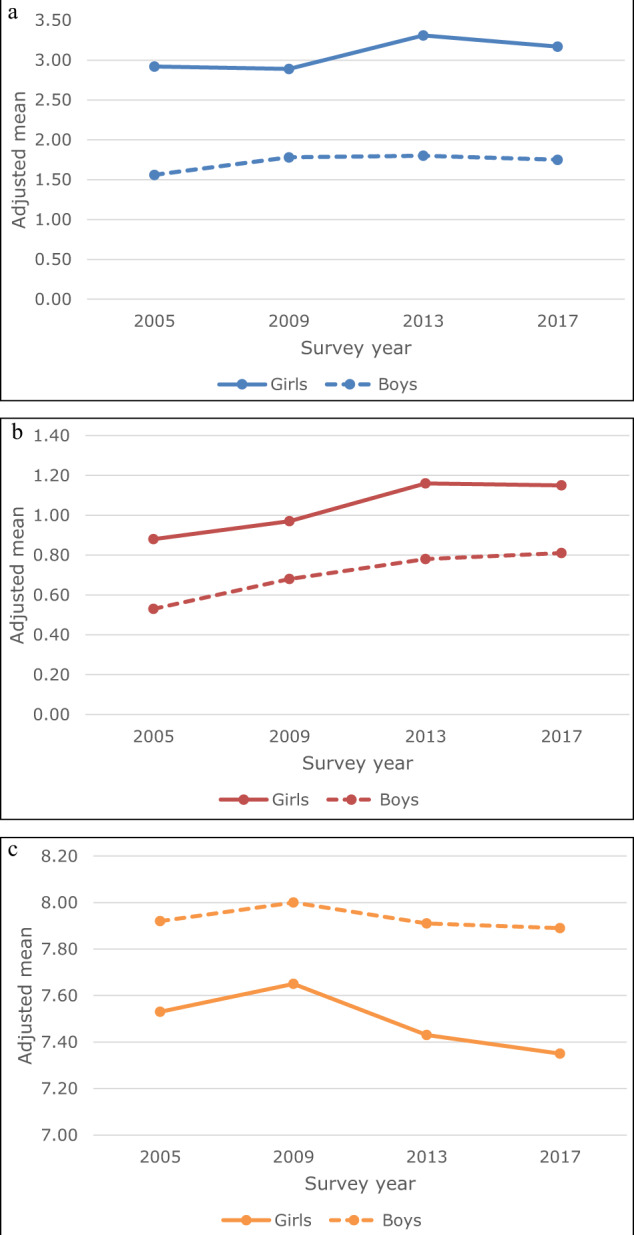
**a** Adjusted means for emotional symptoms (range 0–10) by survey year and gender. **b** Adjusted means for psychosomatic complaints (range 0–4) by survey year and gender. **c** Adjusted means for life satisfaction (range 0–10) by survey year and gender

Table 1 and Fig. 1a–c describe the time trends in the three emotional wellbeing outcomes. Overall, adolescent emotional wellbeing slightly declined over time. Emotional symptoms slightly increased between 2009 and 2013 and stabilised in 2017. Psychosomatic complaints steadily increased steadily between 2005 and 2013 and also stabilised in 2017. Finally, life satisfaction increased between 2005 and 2009, but declined again in 2013 back to the level of 2005. In 2017, life satisfaction declined slightly further to a level that was lower than that of 2005 (but not statistically different from that of 2013). Overall, the largest declines in emotional wellbeing occurred between 2009 and 2013.

**Table 1:** Mean estimates of emotional well-being and explanatory variables between 2005 and 2017 among adolescents (*N* = 5396 for 2005; *N* = 5484 for 2009; *N* = 5187 for 2013; *N* = 5834 for 2017)

Survey year	2005	2009	2013	2017
Emotional well-being				
Emotional symptoms (scale 0–10)	2.23^a^	2.29^a^	2.54^b^	2.44^b^
Psychosomatic complaints (scale 0–4)	0.70^a^	0.82^b^	0.96^c^	0.98^c^
Life satisfaction (scale 0–10)	7.72^a^	7.83^b^	7.68^ac^	7.63^c^
Explanatory variables				
Perceived schoolwork pressure (scale 1–4)	2.00^a^	1.99^a^	2.14^b^	2.29^c^
Communication with mother (scale 1–4)	3.40^a^	3.41^a^	3.36^b^	3.47^c^
Communication with father (scale 1–4)	3.03^a^	3.07^b^	3.06^ab^	3.21^c^
Bullying victimization (scale 1–5)	1.36^a^	1.34^ab^	1.30^b^	1.21^c^

Means are adjusted for gender, age, educational track, family affluence, immigration background, and family structure. Values within the same row with different superscripts differ significantly from one another (*p* < 0.01)

### Perceived Schoolwork Pressure, Parent-Adolescent Communication, and Bullying Victimization: Descriptive Trends

**Fig. 2:**
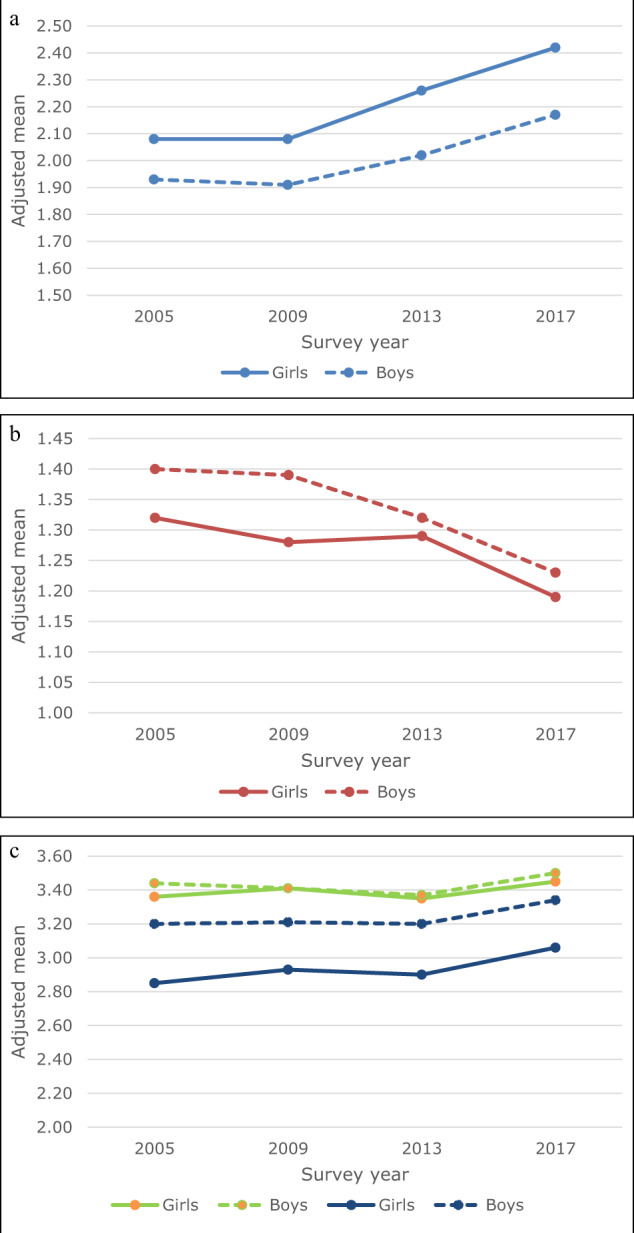
**a** Adjusted means for perceived schoolwork pressure (range 1–4) by survey year and gender. **b** Adjusted means for communication with parents (range 1–4) by survey year and gender. **c** Adjusted means for bullying victimization (range 1–5) by survey year and gender

Table 1 and Fig. 2a–c present the time trends in perceived schoolwork pressure, parent-adolescent communication and bullying victimization. From 2009 onwards, perceived schoolwork pressure increased. Communication with mother was stable between 2005 and 2009, slightly worsened in 2013, and then improved to a level even higher than that of 2005 and 2009 in 2017. Communication with father showed small changes between 2005 and 2013, but improved considerably between 2013 and 2017. Bullying victimization declined, especially between 2013 and 2017.

### Trend Analyses

Results of the trend analyses are shown in Table 2a–c. Emotional symptoms and psychosomatic complaints were higher in 2017, as compared to 2009 and 2005. Life satisfaction in 2017 was lower than in 2009. There were no significant differences in emotional wellbeing (all three indicators) between 2013 and 2017.

**Table 2:** Results of multiple regression analyses examining links with time trends in three indicators of emotional wellbeing in adolescents

	Model 1: Survey year			Model 1 + perceived schoolwork pressure			Model 1 + parent-adolescent communication			Model 1 + bullying victimization			Model 1 + all predictors		
	*B*	*SE*	*p*	*B*	*SE*	*p*	*B*	*SE*	*p*	*B*	*SE*	*p*	*B*	*SE*	*p*
(a) Emotional symptoms															
Survey year															
2017	Ref			Ref			Ref			Ref			Ref		
2013	0.10	0.05	0.053	0.23	0.04	<0.001	−0.03	0.04	0.575	0.04	0.05	0.456	0.07	0.04	0.160
2009	−0.16	0.04	0.002	0.10	0.04	0.037	−0.26	0.04	<0.001	−0.24	0.04	<0.001	−0.08	0.03	0.062
2005	−0.22	0.05	<0.001	0.04	0.04	0.479	−0.34	0.05	<0.001	−0.32	0.05	<0.001	−0.18	0.04	<0.001
Explanatory variables															
Perceived schoolwork pressure				0.87	0.02	<0.001							0.73	0.02	<0.001
Communication with mother							−0.28	0.02	<0.001				−0.21	0.02	<0.001
Communication with father							−0.54	0.02	<0.001				−0.42	0.01	<0.001
Bullying victimization										0.67	0.02	<0.001	0.53	0.02	<0.001
Model fit															
*R*^2^	0.117			0.211			0.180			0.167			0.285		
Wald *F*	219.78			357.65			246.45			281.25			378.82		
(b) Psychosomatic complaints															
Survey year															
2017	Ref			Ref			Ref			Ref			Ref		
2013	−0.01	0.02	0.485	0.03	0.02	0.118	−0.06	0.02	0.002	−0.03	0.02	0.091	−0.03	0.01	0.091
2009	−0.16	0.02	<0.001	−0.07	0.02	<0.001	−0.19	0.02	<0.001	−0.18	0.02	<0.001	−0.13	0.01	<0.001
2005	−0.27	0.02	<0.001	−0.19	0.02	<0.001	−0.31	0.02	<0.001	−0.31	0.02	<0.001	−0.26	0.01	<0.001
Explanatory variables															
Perceived schoolwork pressure				0.28	0.01	<0.001							0.23	0.01	<0.001
Communication with mother							−0.13	0.01	<0.001				−0.11	0.01	<0.001
Communication with father							−0.17	0.01	<0.001				−0.14	0.01	<0.001
Bullying victimization										0.21	0.01	<0.001	0.16	0.01	<0.001
Model fit															
* R*^2^	0.082			0.161			0.150			0.124			0.235		
Wald *F*	136.01			271.27			193.22			187.66			299.34		
(c) Life satisfaction															
Survey year															
2017	Ref			Ref			Ref			Ref			Ref		
2013	0.05	0.04	0.187	−0.03	0.04	0.452	0.17	0.03	<0.001	0.08	0.04	0.020	0.13	0.03	<0.001
2009	0.20	0.04	<0.001	0.07	0.04	0.038	0.28	0.03	<0.001	0.25	0.04	<0.001	0.21	0.03	<0.001
2005	0.10	0.04	0.008	−0.02	0.04	0.452	0.20	0.03	<0.001	0.16	0.04	<0.001	0.14	0.03	<0.001
Explanatory variables															
Perceived schoolwork pressure				−0.42	0.02	<0.001							−0.30	0.02	<0.001
Communication with mother							0.45	0.03	<0.001				0.42	0.02	<0.001
Communication with father							0.39	0.02	<0.001				0.34	0.02	<0.001
Bullying victimization										−0.40	0.02	<0.001	−0.30	0.02	<0.001
Model fit															
* R*^2^	0.08			0.13			0.21			0.12			0.26		
Wald *F*	135.58			175.33			267.81			163.91			275.92		

All models are controlled for age, educational track, family affluence, immigration background, and family structure

When perceived schoolwork pressure was added to the model (Model 1 + perceived schoolwork pressure), trends (2005–2017 and 2009–2017) in emotional symptoms changed from negative to positive. In addition, the difference in emotional symptoms between 2013 and 2017 became significant (from *B* = 0.10 (ns) to *B* = 0.23, *p* < 0.001). This means that, if perceptions of schoolwork pressure had not increased between 2013 and 2017, emotional symptoms might have *decreased* during this period.

Trends in life satisfaction became statistically non-significant after perceived schoolwork pressure was added to the model. For psychosomatic complaints, the trends remained significant, but became less strong. Sobel tests confirmed that the increase in perceived schoolwork pressure significantly explained the decline in wellbeing for all three wellbeing outcomes (*z* for emotional problems = −12.73 (2005) and −12.97 (2009), *p*s < 0.001; *z* for psychosomatic complaints = −12.02 (2005) and −12.22 (2009), *p*s < 0.001; *z* for life satisfaction = 11.25 (2005) and 11.42 (2009), *p*s < 0.001).

When communication with parents was added to the model (Model 1 + parent-adolescent communication), the trends in emotional wellbeing increased slightly in strength. Moreover, for life satisfaction, the insignificant trend between 2013 and 2017 (*B* = 0.05) became significant (*B* = 0.17, *p* < 0.001). This indicates that the improvement in parent-adolescent communication (in 2017, as compared to 2013) may have functioned as a protective factor against the downward trend in life satisfaction. In other words, if there had not been an improvement in parental communication between 2013 and 2017, the downward trend over time in life satisfaction might have persisted into 2017.

When bullying victimization was added to the model (Model 1 + bullying victimization), the trends in emotional wellbeing increased in strength. For example, the *B* of emotional symptoms in 2009 increased from −0.16 to −0.24 (*p*s < 0.001) when bullying victimization was added to the model. This indicates that the decline in bullying victimization may have functioned as a protective factor against the downward trend in emotional wellbeing.

When all explanatory variables were entered in the model together (Model 1 + all predictors), the estimates of survey year approached the estimates of Model 1. This reflects the contrasting directions in which the different explanatory variables accounted for the trends in emotional wellbeing (e.g., increases in schoolwork pressure may have driven the decline in emotional wellbeing, while declines in bullying victimization may have functioned as a protective factor against this decline).

Out of the models in which single predictors were added, the model with schoolwork pressure had the best model fit for emotional symptoms and psychosomatic complaints. For life satisfaction, the model with parent-adolescent communication had the best fit.

### Gender Differences

Girls reported significantly lower emotional wellbeing for all the three outcomes (*B* = 1.35 for emotional symptoms; *B* = 0.33 for psychosomatic complaints; *B* = −0.42 for life satisfaction, *p*s < 0.001; see Fig. 1 for an illustration of these gender differences). Moreover, associations with perceived schoolwork pressure, parent-adolescent communication and bullying victimization were stronger for girls, as compared to boys (*p*s < 0.001; with the exception of *p* < 0.01 for the interaction of gender by perceived schoolwork pressure on life satisfaction). However, trends over time in emotional wellbeing did not differ between boys and girls (i.e., interaction analyses of gender by survey year were not significant). Thus, even though gender differences in emotional wellbeing are considerable, these gender differences in emotional wellbeing have remained stable over time.

### Sensitivity Analyses

Correlation and variance inflation (VIF) analyses were run to test for multicollinearity of the predictors in our model. These analyses indicated that there was no multicollinearity (r ranging from 0.065 to 0.551; the highest correlation being for communication with mother and father; VIF range 1.004–1.872).

## Discussion

Reports on strong declines in young people’s wellbeing in some high-income countries, including Scandinavian countries and the United Kingdom (Bor et al. 2014; Potrebny et al. 2017) and the United States (Mojtabai et al. 2016; Twenge et al. 2018) have raised societal alarms about young people’s wellbeing worldwide. Yet, the generalisability of this decline across other high-income countries is not clear. The present study examined to what extent the declines in emotional wellbeing reported can be generalised to the Netherlands, a country in which adolescents have consistently reported among the highest levels of wellbeing across Europe (e.g., Inchley et al. 2016).

Between 2009 and 2013, adolescent emotional wellbeing in the Netherlands slightly declined. Parallel to this decline in emotional wellbeing, there was a substantial increase in perceived schoolwork pressure. Remarkably, between 2013 and 2017, perceived schoolwork pressure continued to increase, but simultaneous positive developments in the family and peer context (i.e., better parent-adolescent communication and a decline in bullying victimization) may have prevented a further decline in emotional wellbeing.

All in all, this analysis does not feed into the societal alarm regarding the dramatic decline or “crisis” (Gunnell et al. 2018; Twenge 2017) in adolescent wellbeing. The current study is in line with an earlier study on trends in emotional symptoms among Dutch adolescents that identified a small decline between 2009 and 2013 (Duinhof et al. 2015). Similarly, a study among 19–24 year olds showed no increase in mental health problems among Dutch young adults between 2007 and 2017 (Van der Velden et al. 2019). It is important that these findings are shared with adolescents, parents, teachers, and policy makers, as we see a risk in the public debate of an excessive and counterproductive panic regarding the potentially deteriorating mental health of our youth, while there is no strong empirical evidence for it within the Dutch context.

Having said this, as slight changes over time at the population level may mask considerable changes over time in specific high-risk groups, continued attention for the monitoring of emotional wellbeing among adolescents, thereby taking an intersectional approach, is strongly recommended. While this study reported no gender difference in the trends in emotional wellbeing, future research may investigate potential differences in trends for adolescents who are a member of different, or multiple, disadvantaged groups in society (e.g., having low family affluence; having a migration background; Kern et al. 2020).

The current study underlines the need for international comparative research on trends in young people’s wellbeing, as there appear to be strong declines in young people’s wellbeing in some countries (e.g., Potrebny et al. 2017), but not in other countries. The relatively small decline in emotional wellbeing among Dutch adolescents may be related to the relatively positive social context adolescents grow up in. Since the early 21st century, Dutch adolescents have consistently reported very positive relationships with parents and peers, as compared to other high-income countries (Currie et al. 2008a; 2012; Inchley et al. 2016). This positive social context may have provided adolescents in the Netherlands with a good base and with resources to cope with the social stressors of their time, increasing schoolwork pressure being one of them. Future research is however needed to systematically document cross-national differences in trends over time in adolescent emotional wellbeing, and potential explanations of these differences.

While the decline in emotional wellbeing was small, the increase in schoolwork pressure among youth in the Netherlands was substantial and concerning. This may alert professionals in the fields of public health and education to the potential impact of (changes in) school curricula and societal, parental and young people’s expectations regarding their school performance and ambitions. Moreover, potential causes of the increase in schoolwork pressure should be examined, both at a national and international level.

Finally, it is important to understand potential drivers of the positive developments that took place in the family and peer context between 2013 and 2017. The improvement in parent-adolescent communication may be linked to the observation that youth nowadays grow up “less rebellious” and overall have better relationships with their parents (Twenge 2017). The decline in bullying may be the result of the implementation of different anti-bullying policies at schools in the Netherlands (Orobio de Castro et al. 2018). However, as international declines in bullying victimization have been reported between 2002 and 2010 (Chester et al. 2015; Perlus et al. 2014), it would also be relevant to examine international explanations of the decline in bullying victimization.

When it comes to gender differences, this study found that trends over time in emotional wellbeing did not differ for boys and girls. This contradicts findings in some other European countries (Bor et al. 2014) and the United States (Twenge et al. 2018), where the decline in emotional wellbeing was stronger among girls, as compared to boys. While research is needed to understand why the gender gap in emotional wellbeing is increasing in some national contexts but not in others, it is important to underline that the topic of emotional wellbeing deserves attention for both genders.

This study has a number of strengths, such as the use of large and nationally representative datasets, a trend analysis over a relatively long time period of 12 years, and a standard protocol for the data collection across the four study waves. Moreover, while most research on emotional wellbeing is limited in terms of the outcomes used (e.g., only considering psychosomatic complaints (Potrebny et al. 2017) or emotional symptoms (Duinhof et al. 2015)), the current study included three of such outcomes, which strengthens the validity of our conclusions.

The present study is limited by its use of repeated cross-sectional surveys, meaning that causality cannot be inferred. While the increase in perceived schoolwork pressure coincided with a decrease in emotional wellbeing, and the two are negatively associated, this is not sufficient evidence to conclude that the decline in emotional wellbeing was *caused* by an increase in schoolwork pressure. To make such a conclusion, future longitudinal research should investigate whether adolescent emotional wellbeing declines less over time if adolescents experience lower levels of schoolwork pressure. A second limitation concerns the limited availability of wellbeing measures in the HBSC study; depressive symptoms and anxiety were not taken into account. Third, while gender differences are central to this study, our measure of gender (‘are you a boy or a girl?’) has its limitations. Most importantly, it does not reflect the experience of young people whose gender identity does not match these binary categories, nor those for whom the sex assigned at birth does not correspond with their gender identity. Fourth, even though the measure for perceived schoolwork pressure is widely used and has been linked to wellbeing outcomes in previous research as well, it is not clear what this schoolwork pressure exactly entails. For instance, it is not known whether students experience schoolwork pressure related to testing, large amounts of homework, parental or teacher expectations, or whether their perception of schoolwork pressure is a proxy for other types of pressure they experience in the school context (including the trend toward more perfectionism overall; Curran and Hill 2019). Finally, several possible determinants of (trends in) adolescent emotional wellbeing, such as the 2008 economic crisis, young people’s concerns about climate change, increased (mass) migration, increased individualisation in society, and new technologies (Finkenauer et al. 2019), may have contributed to changes in young people’s wellbeing over time, but were not included in the trend analysis due to limited availability of HBSC measures.

## Conclusion

With recent declines being observed in emotional wellbeing among youth in some high-income countries, societal concerns about young people’s emotional wellbeing are on the rise. This study examined trends over time in young people’s wellbeing in the Netherlands, a country in which adolescents have consistently reported among the highest levels of wellbeing across Europe. Emotional wellbeing slightly declined among adolescents in the Netherlands between 2009 and 2013. A substantial increase in schoolwork pressure over time may have driven this decline. Between 2013 and 2017, parent-adolescent communication slightly improved and bullying victimization declined. These positive developments may have prevented a further decline in emotional wellbeing, despite the increasing schoolwork pressure. The increase in schoolwork pressure in particular calls for attention from public health professionals and policy makers. Cross-national comparative research and research into risk groups is needed in order to test the generalisability of our findings.
